# Alcohol or Benzodiazepine Co-involvement With Opioid Overdose Deaths in the United States, 1999-2017

**DOI:** 10.1001/jamanetworkopen.2020.2361

**Published:** 2020-04-09

**Authors:** Marco E. Tori, Marc R. Larochelle, Timothy S. Naimi

**Affiliations:** 1 Department of Medicine, Boston University School of Medicine, Boston Medical Center, Boston, Massachusetts; 2 Clinical Addiction Research and Education Unit, Section of General Internal Medicine, Department of Medicine, Boston University School of Medicine, Boston Medical Center, Boston, Massachusetts; 3 Section of General Internal Medicine, Department of Medicine, Boston University School of Medicine, Boston Medical Center, Boston, Massachusetts; 4 Department of Community Health Sciences, Boston University School of Public Health, Boston, Massachusetts

## Abstract

**Importance:**

The use of benzodiazepines or alcohol together with opioids increases overdose risk, but characterization of co-involvement by predominant opioid subtype is incomplete to date. Understanding the use of respiratory depressants in opioid overdose deaths (OODs) is important for prevention efforts and policy making.

**Objective:**

To assess the prevalence and number of alcohol- or benzodiazepine-involved OODs by opioid subtypes in the United States from 1999 to 2017.

**Design and Setting:**

This repeated cross-sectional analysis used data from the Centers for Disease Control and Prevention Wide-Ranging Online Data for Epidemiologic Research (WONDER) database of all opioid-involved poisoning deaths from January 1, 1999, to December 31, 2017, for the United States. State-level binge drinking prevalence rates for 2015 to 2017 were obtained from the Behavior Risk Factor Surveillance System of the Centers for Disease Control and Prevention, and benzodiazepine prescribing rates for 2012 (most recent available data) were obtained from IMS Health, a commercial database. Data were analyzed from July 10, 2018, to May 16, 2019.

**Main Outcomes and Measures:**

Prevalence of alcohol or benzodiazepine co-involvement for all OODs and by opioid subtype, nationally and by state.

**Results:**

From 1999 to 2017, 399 230 poisoning deaths involved opioids, of which 263 601 (66.0%) were male, and 204 560 (51.2%) were aged 35 to 54 years. Alcohol co-involvement for all opioid overdose deaths increased nonlinearly from 12.4% in 1999 to 14.7% in 2017. By opioid subtype, deaths involving heroin and synthetic opioids (eg, fentanyl; excluding methadone) had the highest alcohol co-involvement at 15.5% and 14.9%, respectively, in 2017. Benzodiazepine co-involvement in all OODs increased nonlinearly from 8.7% in 1999 to 21.0% in 2017. Benzodiazepines were present in 33.1% of prescription OODs and 17.1% of synthetic OODs in 2017. State-level rates of binge drinking were significantly correlated with alcohol co-involvement in all OODs (*r* = 0.34; *P* = .02). State benzodiazepine prescribing rates were significantly correlated with benzodiazepine co-involvement in all OODs (*r* = 0.57; *P* < .001).

**Conclusions and Relevance:**

This study found that alcohol and benzodiazepine co-involvement in opioid-involved overdose deaths was common, varied by opioid subtype, and was associated with state-level binge drinking and benzodiazepine prescribing rates. These results may inform state policy initiatives in harm reduction and overdose prevention efforts.

## Key Points

**Question:**

What is the prevalence of alcohol or benzodiazepine co-involvement in opioid overdose deaths among the opioid subtypes currently and over time?

**Findings:**

This repeated cross-sectional analysis of deaths attributed to opioid overdose identified the co-involvement of alcohol or benzodiazepines as common and increasing, reaching 14.7% for alcohol and 21.0% for benzodiazepines in 2017. Significant correlations occurred between state-level binge drinking rates and alcohol co-involvement and between state-level benzodiazepine prescribing rates and benzodiazepine co-involvement in opioid overdose deaths.

**Meaning:**

Prevalence of respiratory-depressing drugs in opioid overdose deaths is common and increasing and represents a potential target for policy and practice efforts to reduce opioid-related harms.

## Teaser

This cross-sectional analysis assesses the prevalence and number of alcohol- or benzodiazepine-involved opioid overdose deaths by opioid subtypes in the United States from 1999 to 2017.

## Introduction

Poisoning deaths have become the leading cause of unintentional injury death in the United States, killing more than 70 000 people in 2017.^1^ This surge in poisoning deaths is largely made up of opioid overdoses. Increases in opioid overdose deaths (OODs) have been accompanied by the increasing involvement of more potent opioids: first prescription opioids, then heroin, and later illicit fentanyl, now referred to as the *triple wave*.^2^ Characterizing and understanding the effects of and potential solutions for increases in opioid-related mortality have attracted substantial attention^3^; however, polysubstance use among cases of OOD needs further characterization. Specifically, information about the co-involvement of respiratory depressants such as alcohol and benzodiazepines in OODs, overall and by various opioid subtypes, have not been well characterized to date.

Opioid overdose becomes lethal when the central nervous system respiratory drive is suppressed enough to cause hypoxic respiratory failure.^4^ Sedating substances such as alcohol and benzodiazepines can have additive or synergistic effects with opioids on respiratory depression and neuropsychiatric outcomes.^5,6^ Almost half of opioid overdoses in 2016 included alcohol, cocaine, or benzodiazepines.^3^ Polysubstance use is common and often underrecognized but comes with additional health and psychiatric risks,^7^ including accidental overdose. Polysubstance use that includes opioids can take the form of medical co-prescribing, polypharmacy, nonmedical experimentation,^8^ or unintentional contamination.^9^ Limited prevention efforts have been aimed at prescribers and users alike regarding the harms of combining opioids and other substances.^10^

To address the knowledge gaps around the concurrent use of other respiratory depressants with opioids, we characterized the burden of alcohol and benzodiazepine co-involvement in OODs in the United States from January 1, 1999, to December 31, 2017. Moreover, because the predominant opioid subtypes have shifted over time^11^ and may be differentially associated with other substance use, we also assessed differences in co-involvement of alcohol or benzodiazepines among the various opioid subtypes. Finally, we determined correlations of alcohol co-involvement with state-level binge drinking prevalence and of benzodiazepine co-involvement with state-level benzodiazepine prescribing to explore whether population-based prevention strategies might influence co-involvement of respiratory depressants in OODs.

## Methods

### Data Source

The study protocol followed the Strengthening the Reporting of Observational Studies in Epidemiology (STROBE) reporting guideline for cross-sectional studies. The Boston University institutional review board determined this study to be nonhuman subjects research and did not require approval or informed consent.

We used the multiple-cause-of-death files from the National Vital Statistics System of the Centers for Disease Control and Prevention Wide-Ranging Online Data for Epidemiologic Research (WONDER) database^12^ to study all opioid-involved poisoning deaths from 1999 to 2017. Overdose and drug poisoning deaths were defined using the *International Statistical Classification of Diseases, Tenth Revision, Clinical Modification* (*ICD-10-CM*)^13^ codes X40 to X44, X60 to X64, X85, and Y10 to Y14 for underlying cause of death.^14^ The cohort of drug poisoning death cases was narrowed to opioid-involved deaths by including the opioid *ICD-10-CM* codes (T40.0 [opium], T40.1 [heroin], T40.2 [other opioids], T40.3 [methadone], T40.4 [other synthetic narcotics], and T40.6 [other and unspecified narcotics]) in the multiple-cause-of-death listings. The *ICD-10-CM* code T40.2 for other opioids refers to natural and semisynthetic opioids, including morphine, hydrocodone, oxycodone, and codeine, which we will refer to as prescription opioids. The *ICD-10-CM* code T40.4 for other synthetic narcotics refers to fentanyl, fentanyl analogues, and tramadol and explicitly excludes methadone.^15^ Some death certificates include multiple opioid subtype codes to reflect a poly-opioid overdose; therefore, categories of deaths by subtype were not mutually exclusive. Benzodiazepine or alcohol involvement, determined by inclusion of those substances on the death certificate as multiple causes of death, was selected for using *ICD-10-CM* codes T42.4 (benzodiazepines), T51.0 (ethanol), and T51.9 (alcohol, unspecified).

### Analytical Strategy

Data were analyzed from July 10, 2018, to May 16, 2019. Using descriptive statistics, we conducted a repeated cross-sectional analysis of annual OODs and the proportion with benzodiazepine and alcohol co-involvement, stratified by opioid subtype. We then performed a geographic subanalysis of alcohol and benzodiazepine co-involvement for all OODs in all 50 US states and the District of Columbia using combined 2015-2017 data (the 3 most recent years of available mortality data). A Pearson correlation test was used to compare the co-involvement prevalence rates of alcohol and benzodiazepines in each state. We also ran correlation testing between the state-level prevalence of alcohol co-involvement in OODs and state-level prevalence of binge drinking in 2015 to 2017 using data from the Behavior Risk Factor Surveillance System,^16^ which defines binge drinking as 5 or more standard drinks on 1 occasion for men and 4 or more for women, self-reported in the last month. Finally, we assessed the correlation between benzodiazepine co-involvement in all OODs in 2012 and state-level benzodiazepine prescribing rates (the most recent data available) in 2012.^17^ All analyses were performed with Microsoft Excel (Professional Plus, version 2013). One-sided *P* < .05 indicated significance.

## Results

### Trends in Alcohol and Benzodiazepine Co-involvement

**Figure 1.**
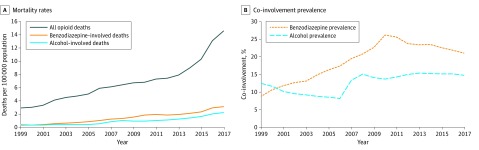
Co-involvement of Alcohol and Benzodiazepine in All Opioid Overdose Deaths Data are from the United States, 1999 to 2017.

From 1999 to 2017, 399 230 poisoning deaths involving opioids were reported, of which 135 629 (40.0%) involved females; 263 601 (66.0%), males; and 204 560 (51.2%), persons aged 35 to 54 years. During the study period, the prevalence of co-involvement increased overall but nonlinearly for alcohol and benzodiazepines in OODs. Alcohol co-involvement persisted near 15% for all opioid overdoses since 2008, a change from 12.4% in 1999 to 14.7% in 2017. Benzodiazepine co-involvement increased from 8.7% in 1999 to 26.2% in 2010 and declined to 21.0% in 2017 (Figure 1). Overall mortality rates for opioid overdoses involving benzodiazepines increased 10.3-fold, and those involving alcohol increased 5.5-fold from 1999 to 2017; OOD rates increased 5.0-fold during the same period.

**Figure 2.**
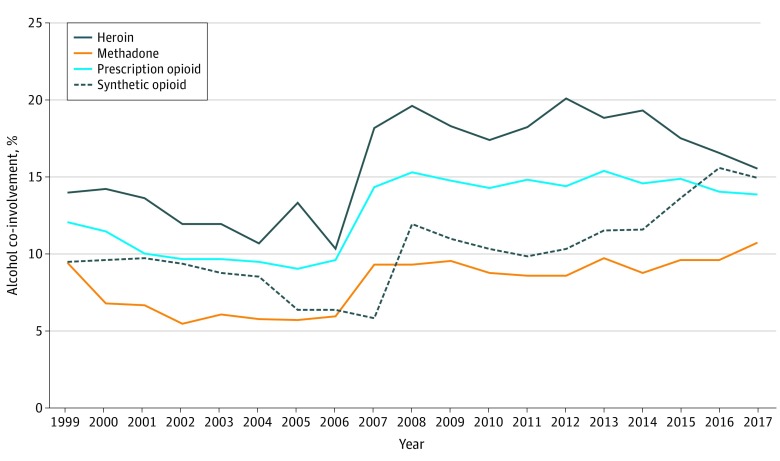
Alcohol Co-involvement Among Overdose Deaths by Opioid Type Data are from the United States, 1999 to 2017.

Trends for alcohol co-involvement differed by opioid subtype (Figure 2). Alcohol co-involvement in heroin overdose deaths ranged from 10.3% to 20.1% during the study period and was 15.5% in 2017, representing 0.7 deaths per 100 000 persons in 2017. Alcohol co-involvement among OODs involving synthetic opioids (eg, fentanyl) increased from 11.5% in 2013 to 14.9% in 2017 (1.3 deaths per 100 000 persons in 2017). For OODs involving prescription opioids (eg, oxycodone), alcohol co-involvement ranged from 9.0% to 15.4% for the study period but more narrowly from 14.3% to 15.4% from 2007 to 2017 and represented 0.7 deaths per 100 000 persons in 2017. Methadone and alcohol co-involvement remained at less than 10% until 2017 but only represented 0.1 deaths per 100 000 persons that year.

**Figure 3.**
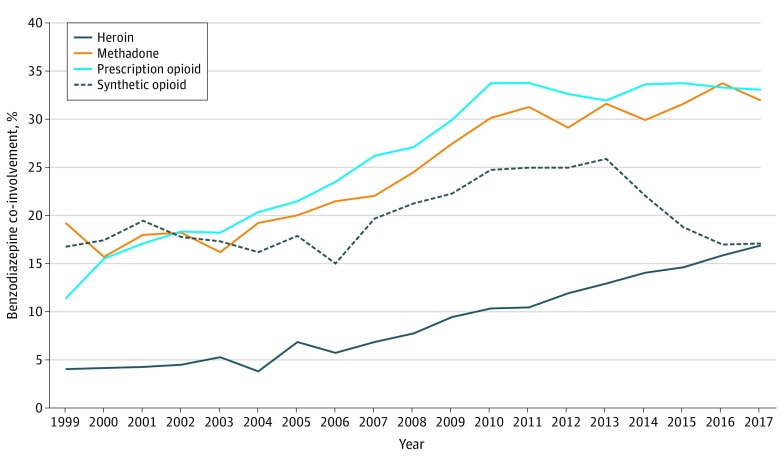
Benzodiazepine Co-involvement Among Overdose Deaths by Opioid Subtype Data are from the United States, 1999 to 2017.

Benzodiazepine co-involvement proportions in OODs increased among all opioid subtypes from 1999 to 2017 (Figure 3). For OODs involving heroin, co-involvement increased from 4.0% in 1999 to 17.1% in 2017 (0.8 deaths per 100 000 persons in 2017). For OODs involving synthetic opioids, benzodiazepine co-involvement ranged from 15.0% to 25.9%, representing 1.5 deaths per 100 000 persons in 2017. Benzodiazepine co-involvement in methadone OODs increased from 15.6% of all involved deaths in 2000 to 32.0% in 2017, representing 0.3 deaths per 100 000 persons. Prescription opioid OODs had 15.6% benzodiazepine co-involvement in 2000 and increased to 33.1% in 2017, representing 1.5 deaths per 100 000 persons that year.

### Current Co-involvement in 2015-2017 by Opioid Subtype, Sex, and State

During 2015 to 2017, the 3 most recent years of available data, synthetic opioids excluding methadone contributed to 46.7% of all OODs, whereas prescription opioids contributed to 33.9% (Table). Overall, 67.1% of all OODs occurred in men. Women constituted 42.6% of OODs involving prescription opioids and 28.8% of OODs involving synthetic opioids.

**Table.** OODs With Alcohol and Benzodiazepine Co-involvement Prevalence, by Opioid Subtype and Sex^a^

Opioid Type	No. (%) of OODs		
	All (n = 122 940)	Men (n = 82 506)	Women (n = 40 434)
All OODs			
Alcohol co-involvement^b^	18 419 (15.0)	14 178 (17.2)	4241 (10.5)
Benzodiazepine co-involvement^c^	26 728 (21.7)	15 837 (19.2)	10 891 (26.9)
Heroin deaths			
All	43 940 (35.7)	33 229 (40.1)	10 711 (26.5)
Alcohol co-involvement^b^	7245 (16.5)	6013 (18.1)	1232 (11.5)
Benzodiazepine co-involvement^c^	6972 (15.9)	4895 (14.7)	2077 (19.4)
Methadone deaths			
All	9868 (8.0)	5817 (7.1)	4051 (10.0)
Alcohol co-involvement^b^	984 (10.0)	690 (11.9)	294 (7.3)
Benzodiazepine co-involvement^c^	3202 (32.4)	1835 (31.5)	1367 (33.7)
Prescription opioid deaths^d^			
All	41 709 (33.9)	23 932 (29.0)	17 777 (44.0)
Alcohol co-involvement^b^	5936 (14.2)	4123 (17.2)	1813 (10.2)
Benzodiazepine co-involvement^c^	13 905 (33.3)	7499 (31.3)	6406 (36.0)
Synthetic opioid deaths^e^			
All	57 459 (46.7)	40 919 (49.6)	16 540 (40.9)
Alcohol co-involvement^b^	8584 (14.9)	6787 (16.6)	1767 (10.7)
Benzodiazepine co-involvement^c^	9978 (17.4)	6356 (15.5)	3622 (21.9)

Abbreviation: OOD, opioid overdose death.

^a^ Data are from the United States, 2015 to 2017. Subtype deaths total greater than all opioids deaths reported because the subtypes are not mutually exclusive.

^b^ Indicates presence of *International Statistical Classification of Diseases, Tenth Revision, Clinical Modification *(*ICD-10-CM*) code T51.0 (ethanol) or T51.9 (alcohol, unspecified) on death certificate.

^c^ Indicates presence of *ICD-10-CM* code T42.4 (benzodiazepine) on death certificate.

^d^ Natural and semisynthetic opioids including hydrocodone, oxycodone, and morphine (*ICD-10-CM* code T40.2).

^e^ Explicitly excludes methadone and includes fentanyl and trazodone (*ICD-10-CM* code T40.4).

The prevalence of alcohol co-involvement in all OODs was 17.2% for men and 10.5% for women, whereas benzodiazepine co-involvement was 19.2% for men and 26.9% for women. Similar alcohol co-involvement proportions for both sexes were observed in the heroin, prescription, and synthetic opioid subtypes. Different benzodiazepine co-involvement prevalence was observed between the prescription opioid and synthetic opioid subtypes for both sexes, with higher co-involvement seen in the prescription opioid subtype (31.3% co-involvement in men and 36.0% in women) (Table).

Co-involvement prevalence of alcohol or benzodiazepines varied widely by state during 2015 to 2017 (eTable in the Supplement). Alcohol co-involvement in all OODs ranged from 7.4% in Mississippi to 28.7% in the District of Columbia, with a median of 13.3%. Benzodiazepine co-involvement had a wider range than alcohol, ranging from 5.4% in Delaware to 45.5% in Arkansas, with a median of 22.0%. For all OODs from 2015 to 2017, no correlation was found between the alcohol and benzodiazepine co-involvement prevalence in each state (*r* = 0.08; *P* = .56).

### Association of Co-involvement With State-Level Binge Drinking and Benzodiazepine Prescribing

**Figure 4.**
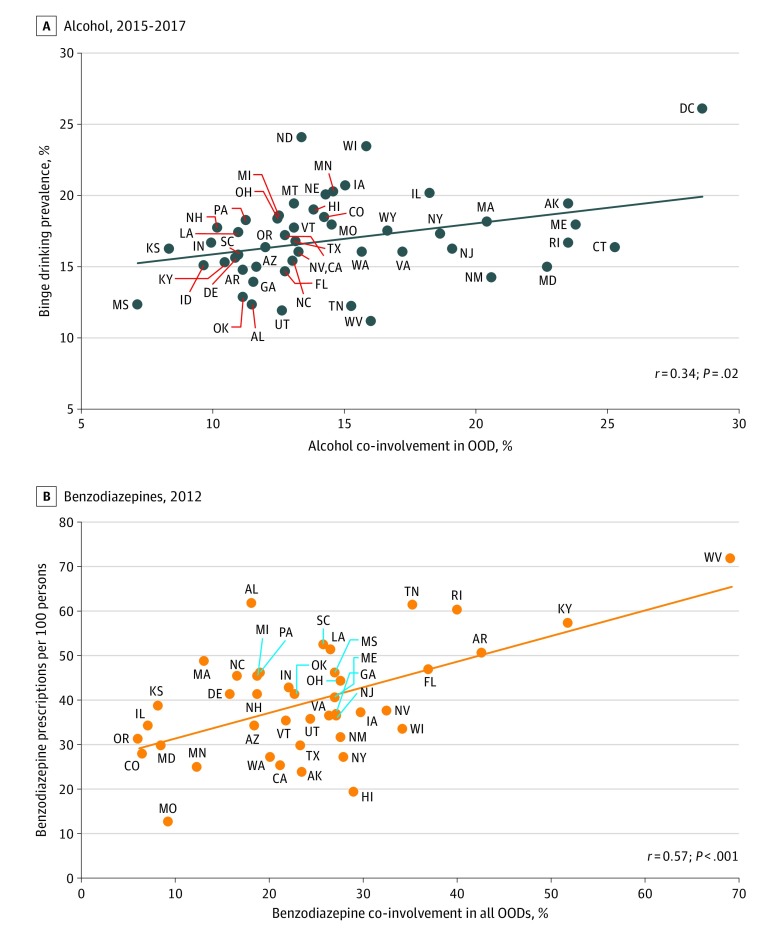
State-Level Association of Substance Use with Co-involvement in All Opioid Overdose Deaths (OODs) Connecticut, the District of Columbia, Idaho, Montana, Nebraska, Nevada, and Wyoming did not have enough incidence of benzodiazepine co-involvement in all OODs for the year 2012. South Dakota did not have enough incidence of benzodiazepine or alcohol co-involvement.

Across states, the prevalence of self-reported alcohol binge drinking was positively correlated with the prevalence of alcohol co-involvement in all opioid overdose deaths for 2015 to 2017 (*r* = 0.34; *P* = .02) (Figure 4). There was also a positive correlation between the prevalence of benzodiazepine co-involvement in all OODs for 2012 and state benzodiazepine prescribing rates (*r* = 0.57; *P* < .001) per 100 persons from 2012 commercial pharmacy data (Figure 4).

## Discussion

We found that alcohol and benzodiazepine co-involvement in opioid overdose deaths is common, and the prevalence and mortality rates of each co-involved substance has increased during the past 2 decades. Notably, prevalence of benzodiazepine co-involvement in OODs peaked in 2010 and has decreased as OOD increases have become attributable to first heroin and then illicitly sourced fentanyl. At the same time, the prevalence of alcohol co-involvement has remained steady. By 2017, alcohol and benzodiazepine co-involvement in all opioid deaths occurred at similar frequencies. Data from 2015 to 2017 show alcohol co-involvement was relatively higher in the heroin and synthetic (largely illicit fentanyl) subtypes compared with the prescription opioid subtype, and conversely, benzodiazepine co-involvement was higher in the prescription opioid subtype. Alcohol co-involvement was more prevalent in men across all subtypes, whereas benzodiazepine co-involvement was more common in women, consistent with known use patterns of those substances.^18,19^

When analyzed by opioid subtype, the co-involvement prevalence has changed over time. Alcohol co-involvement was stable from 2013 to 2017 for all OODs; however, subtype trends showed alcohol co-involvement decreasing in the heroin subtype and increasing in the synthetic subtype during this time period. Alcohol co-involvement in OODs for all opioid subtypes increased since 1999, although changes in reporting of deaths and underreporting^20^ of alcohol use may explain this trend. Benzodiazepine co-involvement for all OODs appears to have decreased since 2010, possibly driven by the decreasing prevalence in the synthetic opioid subtype, which as an involved subtype has increased in mortality incidence. Benzodiazepine co-involvement remains prevalent in nearly one-third of deaths involving prescription opioids.

We specifically looked at alcohol and benzodiazepines because they are respiratory depressants and have been targets of public health campaigns.^21,22^ Benzodiazepines and alcohol both suppress the respiratory drive and have synergistic effects with opioids that likely increase the risk of a fatal overdose when substances are used concurrently. Despite black box warnings on benzodiazepine and opioid prescriptions cautioning against combination use and consumption with alcohol,^22,23,24^ our study demonstrates that co-use is common. Access to benzodiazepines, unlike opioids and alcohol, remains largely under the control of physicians,^25^ and benzodiazepine and opioid co-prescribing rates have quadrupled from 2003 to 2015, mainly occurring in the primary care setting.^26^ Addressing co-prescribing by physicians remains an important policy initiative, although prescription opioids make up a minority of overdose deaths.

Although co-involvement prevalence is high in prescription OODs, fentanyl-involved OODs made up nearly half of all OODs in the last 3 years of available data. Additional studies are needed to understand polysubstance use trends, including alcohol, among those who use fentanyl,^27^ because illicitly manufactured fentanyl remains the driving force in overdose deaths and is responsible for the sharp increase in deaths since 2013. Fentanyl causes respiratory depression and death more rapidly than other common opioids, possibly decreasing the importance of additional respiratory depressants as a contributor to death.

Both alcohol use and benzodiazepine use are modifiable risk factors for overdose. We believe confronting unhealthy alcohol use and access to alcohol is an important state and national policy initiative because the disease burden of alcohol use is greater than that of opioids.^28^ Examples of effective population-based strategies to reduce binge drinking include increasing alcohol taxes, maintaining restrictions on alcohol outlet density and hours of sale, and enforcing minimum drinking age laws.^29^ Innovations to decrease benzodiazepine co-prescribing are needed. One study of electronic medical alerts^30^ demonstrated a modest effect, but more work is needed on this front. On a population level, we observed a correlation between state binge drinking prevalence and alcohol co-involvement prevalence in OODs. Individuals who binge drink are nearly twice as likely to misuse prescription opioids, and higher frequency of binge drinking corresponds to higher prevalence of opioid misuse.^31^ The co-use of alcohol and illicit opioids, specifically heroin and illicitly manufactured fentanyl, deserves further research at the individual and population level. Further understanding alcohol consumption patterns and benzodiazepine use among individuals who use opioids may refine harm reduction messaging and provide promising avenues for prevention of overdoses.

To our knowledge, our study is the first to characterize alcohol and benzodiazepine co-involvement prevalence completely by opioid subtype across the scope of the opioid epidemic. Other studies^5,32,33^ affirm our findings that co-involvement with alcohol and benzodiazepines differs among opioid subtypes, but they do not examine trends over the trajectory of the epidemic. Using toxicology data, 1 study^32^ saw a prevalence of alcohol co-involvement among opioid subtypes similar to that of our study, but these are data from the first wave of the epidemic and do not include heroin or illicit fentanyl. In a study of opioid-related deaths limited to Massachusetts, Barocas et al^33^ show a 21% benzodiazepine-only co-intoxication, 18% alcohol-only co-intoxication, and 42% polysubstance involvement for all OODs. A study by Gomes et al^5^ demonstrated higher alcohol co-involvement (22.3%) in all OODs and a mean postmortem blood alcohol concentration of 0.14 mg/dL in Ontario, Canada, although an analysis by opioid subtype was not performed. Two studies^34,35^ show a benzodiazepine co-involvement proportion at 60% in toxicological postmortem analysis, inconsistent with any data in this study.

### Limitations

Our study has several limitations. First, the Centers for Disease Control and Prevention WONDER database relies on death certificate data, which are known to have misclassifications and imperfect conversion of deaths into standard *ICD-10-CM* codes^15^ and vary by state reporting practices,^36^ missing drug information, or drug misidentification. In 2011, roughly 20% of drug poisoning deaths did not have specific drugs listed,^37^ although that number improved to 12% in 2017^38^; therefore, the numbers reported herein are likely underestimated. Standards for reporting suspected opioid overdose deaths changed during the study period and have likely increased recognition of co-intoxicants in more recent years. The National Association of Medical Examiners Guidelines for Opioid Drug Overdoses,^39^ first published in 2013, specifically recommends testing for benzodiazepines but does not specifically address alcohol. Alcohol is an underreported contributor to mortality and is often excluded from drug-related deaths.^20,40,41^

Variation in year-to-year reporting practices makes trend interpretation difficult. The change in alcohol co-involvement seen from 2006 to 2007 may be associated with increases in state reporting practice. Eight additional states reported data in 2007 compared with 2006, and 8 other states reported 481 additional alcohol–co-involved deaths in 2007. States’ ability to specify at least 1 drug at the time of death ranged from 54.7% to 99.3% in 2017,^42^ limiting comparison between states. The observed state-level correlations between co-involvement and binge drinking or benzodiazepine prescribing are not causal and may be prone to observational biases. These findings suggest that further research is warranted. Because the database does not offer individual-level data, we are unable to identify the number of deaths involving opioids, benzodiazepines, and alcohol, which may offer additional insight into deaths from polysubstance use. In addition, we cannot look at ethanol or drug toxicology levels, which assist in dose-response assessment. Further research on polysubstance opioid overdose should focus on toxicology data and risk factors for overdose, especially in the changing landscape of polysubstance use.^43^

## Conclusions

This study found that the proportion and rates of OODs involving alcohol and benzodiazepines have increased during the past 20 years. Proportions of co-involvement vary by opioid subtype recently and over time, highlighting changing aspects of the ongoing epidemic. Alcohol remains an important and underrecognized cointoxicant in overdose deaths. Benzodiazepines remain especially prevalent in prescription OODs, and their changing involvement in heroin and synthetic OODs warrants further evaluation. Associations between OODs, binge drinking, and benzodiazepine prescribing rates may help identify risk factors and prevention strategies for opioid overdoses and OODs on a population level.

## Notes

Supplement. **eTable.** State-Specific Rates of Alcohol and Benzodiazepine Co-involvement, 2015-2017
