# Supplementary material for: Alcohol or Benzodiazepine Co-involvement With Opioid Overdose Deaths in the United States, 1999-2017
Source: JAMA Netw Open. 2020 Apr 9;3(4):e202361. doi: 10.1001/jamanetworkopen.2020.2361 (PMC7146101; doi:10.1001/jamanetworkopen.2020.2361)
Supplement: Supplement. — eTable. State-Specific Rates of Alcohol and Benzodiazepine Co-involvement, 2015-2017 [file jamanetwopen-3-e202361-s001.pdf]

Supplementary Online Content

Tori ME, LaRochelle MR, Naimi TS. Alcohol or benzodiazepine co-involvement with opioid overdose deaths in the United States, 1999-2017. *JAMA Netw Open*. 2020;3(4):e202361. doi:10.1001/jamanetworkopen.2020.2361

eTable. State-Specific Rates of Alcohol and Benzodiazepine Co-involvement, 2015-2017

This supplementary material has been provided by the authors to give readers additional information about their work.

©2020 Tori ME et al. *JAMA Network Open*.

eTable. State-Specific Rates of Alcohol and Benzodiazepine Co-involvement, 2015-2017

	All Opioid Overdose Deaths	Crude Death Rate per 100,000	Alcohol Co-involvement Deaths	Alcohol Co-involvement Prevalence	Percent Binge Drinking[a], 2015-2017	Benzodiazepine Co-involvement Deaths	Benzodiazepine Co-involvement Prevalence	Benzodiazepine Prescription per 100 persons[b], 2012
Alabama	1047	7.2	124	11.1%	11.6%	208	19.1%	61.9
Alaska	282	12.7	71	24.5%	20.4%	70	23.4%	24.0
Arizona	2368	11.4	289	11.8%	14.2%	548	22.4%	34.3
Arkansas	560	6.2	65	10.9%	14.1%	260	45.5%	50.8
California	6229	5.3	869	13.3%	16.5%	1354	20.7%	25.4
Colorado	1609	9.7	238	14.2%	17.6%	263	16.2%	28.0
Connecticut	2495	23.2	639	25.4%	16.9%	746	29.5%	46.2
Delaware	537	18.8	60	10.8%	14.9%	29	5.4%	41.5
District of Columbia	551	26.9	160	28.7%	27.2%	39	6.7%	38.4
Florida	7881	12.7	1048	13.0%	15.6%	1774	22.2%	46.9
Georgia	2790	9	332	11.6%	15.3%	755	26.4%	37.0
Hawaii	192	4.5	28	14.6%	18.9%	68	32.8%	19.3
Idaho	312	6.2	31	9.3%	14.1%	56	17.6%	29.1

©2020 Tori ME et al. *JAMA Network Open*.

Illinois	5530	14.4	1041	18.4%	20.2%	1028	18.2%	34.2
Indiana	2505	12.6	259	9.6%	15.7%	539	20.8%	42.9
Iowa	559	5.9	89	14.7%	19.8%	142	24.0%	37.3
Kansas	440	5	41	7.5%	15.6%	90	19.8%	38.9
Kentucky	3034	22.8	326	10.4%	15.3%	1042	33.5%	57.4
Louisiana	1048	7.5	120	11.0%	17.3%	230	21.5%	51.4
Maine	899	22.5	218	23.8%	17.6%	249	27.4%	40.7
Maryland	4893	27.1	1127	22.7%	14.2%	327	6.6%	29.9
Massachusetts	5453	26.6	1133	20.5%	17.7%	799	14.4%	48.8
Michigan	5104	17.1	651	12.4%	18.5%	1016	19.5%	45.5
Minnesota	1156	7	173	14.7%	19.5%	222	19.1%	24.9
Mississippi	515	5.7	38	7.4%	11.9%	155	29.3%	46.2
Missouri	2558	14	384	14.5%	16.7%	220	8.2%	12.6
Montana	128	4.1	17	13.3%	19.8%	13	10.2%	33.7
Nebraska	158	2.8	24	13.9%	19.5%	67	41.1%	35.0
Nevada	1239	14	183	13.4%	14.2%	382	29.1%	37.5
New Hampshire	1241	31	128	10.0%	16.5%	94	7.5%	41.2
New Jersey	4240	15.8	832	19.2%	16.0%	963	22.2%	36.5

©2020 Tori ME et al. *JAMA Network Open*.

New Mexico	1032	16.5	227	21.1%	12.9%	350	32.7%	31.5
New York	8399	14.1	1607	18.8%	16.7%	2399	28.0%	27.3
North Carolina	4630	15.2	601	12.7%	13.8%	1387	29.5%	45.3
North Dakota	123	5.4	17	13.0%	24.1%	16	13.0%	31.1
Ohio	10604	30.4	1350	12.4%	18.2%	1579	14.4%	41.3
Oklahoma	1259	10.7	145	10.8%	13.1%	281	22.0%	44.5
Oregon	987	8	122	11.9%	16.6%	93	9.1%	31.4
Pennsylvania	6145	16	701	11.2%	17.0%	1080	17.3%	46.1
Rhode Island	810	25.5	193	23.7%	16.0%	205	25.2%	60.2
South Carolina	1931	13	221	11.1%	15.2%	590	29.5%	52.6
South Dakota[c]	104	4	Suppressed	--	16.9%	Suppressed	--	28.0
Tennessee	3493	17.5	552	15.3%	10.3%	1462	40.6%	61.4
Texas	4120	4.9	550	12.9%	15.9%	1186	28.3%	29.8
Utah	1370	15	179	12.8%	11.6%	484	34.5%	35.9
Vermont	294	15.7	40	13.3%	17.2%	28	9.2%	35.5
Virginia	3191	12.6	556	17.3%	16.3%	523	16.3%	36.4

©2020 Tori ME et al. *JAMA Network Open*.

Washington	2143	9.8	347	15.8%	16.1%	382	17.5%	27.1
West Virginia	2195	40	367	15.6%	10.6%	924	40.4%	71.9
Wisconsin	2414	13.9	389	15.8%	22.9%	635	25.8%	33.4
Wyoming	143	8.2	25	16.1%	16.0%	27	18.9%	24.1

[a]definition of binge drinking: >4/5 drinks in one occasion for women/men respectively within the last 30 days and BRFSS source

[b]benzodiazepine state prescribing rates obtained from reference 17 in manuscript text

[c]South Dakota does not have high enough incidence (>10 cases) to report values.

©2020 Tori ME et al. *JAMA Network Open*.
